# The Effect of Endotracheal Tube (ETT) Tip Position on Lung Aeration in Term and Preterm Neonates: A Comparative Analysis

**DOI:** 10.7759/cureus.79529

**Published:** 2025-02-23

**Authors:** Muhammad Azeem Khan, Faraz Ahmed, Sana Memon, Lamia Batool Rizvi, Hina Mohsin, Aisha Afzal, Sher Wali Khan, Syed Rehan Ali

**Affiliations:** 1 Neonatology, Sindh Institute of Child Health and Neonatology, Karachi, PAK; 2 Medicine, Ziauddin University, Karachi, PAK; 3 Radiology, Sindh Institute of Child Health and Neonatology, Karachi, PAK; 4 Research and Development, Sindh Institute of Child Health and Neonatology, Karachi, PAK; 5 Neonatology, Indus Hospital and Health Network, Karachi, PAK

**Keywords:** ett, lung aeration, neonates, nicu, preterm

## Abstract

Background: Proper endotracheal tube (ETT) position is crucial for neonatal lung aeration. The purpose of this study is to determine the effect of ETT tip position on lung aeration in term and preterm infants.

Methods: This retrospective chart study involved neonates who were admitted to the Neonatal Intensive Care Unit (NICU) and intubated, and it was carried out from February 2023 to July 2023 at the Sindh Institute of Child Health and Neonatology. ETT tip position was analyzed, and chest x-rays (CXRs) were obtained within four hours of intubation. On a CXR, lung expansion evident to eight or eight and a half ribs was considered adequate lung inflation/aeration; fewer than eight ribs were considered poor lung inflation/aeration. To ascertain relationships between ETT tip location and lung aeration, data were examined using the chi-square test in SPSS version 26 (IBM Corp., Armonk, NY).

Results: Out of 149 neonates, 105 (70.5%) were preterm and 44 (29.5%) were term. Optimal lung aeration was observed in 124 neonates (83.2%). The ETT tip was positioned at T1-T2 in 86 neonates (57.7%) and at T3-T4 in 63 neonates (42.3%). For term neonates, those with the ETT tip at T1-T2 exhibited significantly higher rates of optimal lung aeration (72.2%) compared to those with the tip at T3-T4 (27.8%, p = 0.019). Conversely, no significant difference in lung aeration was noted among preterm neonates based on ETT position (p = 0.745).

Conclusions: In conclusion, our study found a significant association between ETT tip positioning at T1-T2 and optimal lung aeration in term neonates. This suggests that precise ETT placement may play an important role in achieving better lung aeration in term infants, while slight positional deviations may be less impactful for lung aeration in preterm neonates. These findings may guide NICU protocols to consider gestational age when tailoring ventilation strategies, emphasizing the importance of anatomical and physiological differences in neonatal respiratory care.

## Introduction

While non-invasive ventilation has become increasingly preferred in neonatal respiratory management, endotracheal intubation (EI) remains a critical intervention for cases where non-invasive support is insufficient, particularly in severe neonatal respiratory distress [1]. It is performed either as an emergency intervention or electively for various indications, such as failure of mask airway control, airway abnormalities, prematurity, and surfactant administration in premature neonates [2].

One important aspect that has been found to significantly affect pulmonary outcomes in critically unwell newborns is the positioning of the endotracheal tube (ETT). According to Thayyil et al., in ventilated premature newborns, the location of the ETT tip is independently associated with adverse outcomes, including poorer survival rates and non-uniform lung aeration [3]. They found that newborns with suboptimal ETT positioning had considerably more non-uniform lung aeration than infants with optimal ETT positioning, highlighting the significance of accurate ETT deployment.

Similarly, Maine et al. found that malpositioned ETTs, particularly those near or below the carina, were associated with uneven lung expansion and inefficient surfactant administration in newborns [4]. These findings are consistent with previous studies by Greenough, who found an obvious association between ETT malpositioning and the development of asymmetrical pulmonary interstitial emphysema related to barotrauma [5].

Chest radiographs remain the gold standard for confirming ETT placement, despite their cost and the need for repeated imaging [6-8]. The capacity to quickly diagnose and address ETT malposition is critical for improving newborn outcomes and avoiding mechanical ventilation-related complications.

Given the critical importance of accurate ETT positioning, this study aims to explore the association between ETT tip position and optimal lung aeration in neonates admitted to the NICU at a tertiary care center in Karachi, Pakistan. By correlating ETT placement with lung aeration, this research seeks to contribute to the optimization of neonatal care and the reduction of morbidity associated with ETT malposition.

## Materials and methods

This retrospective chart review was conducted over a six-month period from February 2023 to July 2023 at the Neonatal Intensive Care Unit (NICU) at the Sindh Institute of Child Health and Neonatology in Karachi, Pakistan. The study was approved by the institutional review board (IRB) of the Sindh Institute of Child Health and Neonatology (IRB number: SICHN/Ex-004/2024). Non-probability consecutive sampling was utilized in a cross-sectional analytical strategy to select participants from existing records. Inclusion criteria comprised neonates who were intubated and admitted to the NICU, those receiving synchronized intermittent mandatory ventilation (SIMV), intermittent positive pressure ventilation (IPPV), or high-frequency oscillation (HFO) ventilation, and those whose parents or guardians had previously provided informed consent for a chest x-ray (CXR) to be taken within four hours of intubation. Exclusion criteria included neonates with congenital malformations, chromosomal abnormalities, hydrops fetalis, nasal intubation, aberrant esophageal intubation, and unsatisfactory CXR images due to exposure or rotation issues.

A standardized Performa (Performa Software Inc., Rockville, MD) was used for data collection, capturing demographic information such as gender, weight, admission date, gestational age, and size appropriate for gestational age. Two sets of CXRs were prepared to ensure unbiased evaluation. In the first set, the position of the ETT was obscured using photo editing software, allowing the radiologists to assess the lung fields exclusively for inflation. In the second set, the lung fields were masked to enable the radiologists to focus solely on evaluating the ETT tip position. Both sets of CXRs were randomized prior to their evaluation to eliminate any potential bias in interpretation.

All CXRs were analyzed by experienced pediatric radiologists with at least five years of expertise. The radiologists were blinded to the study’s objectives, as well as to the original ETT positions and lung field characteristics. This blinding was further ensured by the masking of the ETT position and lung fields in the respective sets of CXRs.

**Figure 1:**
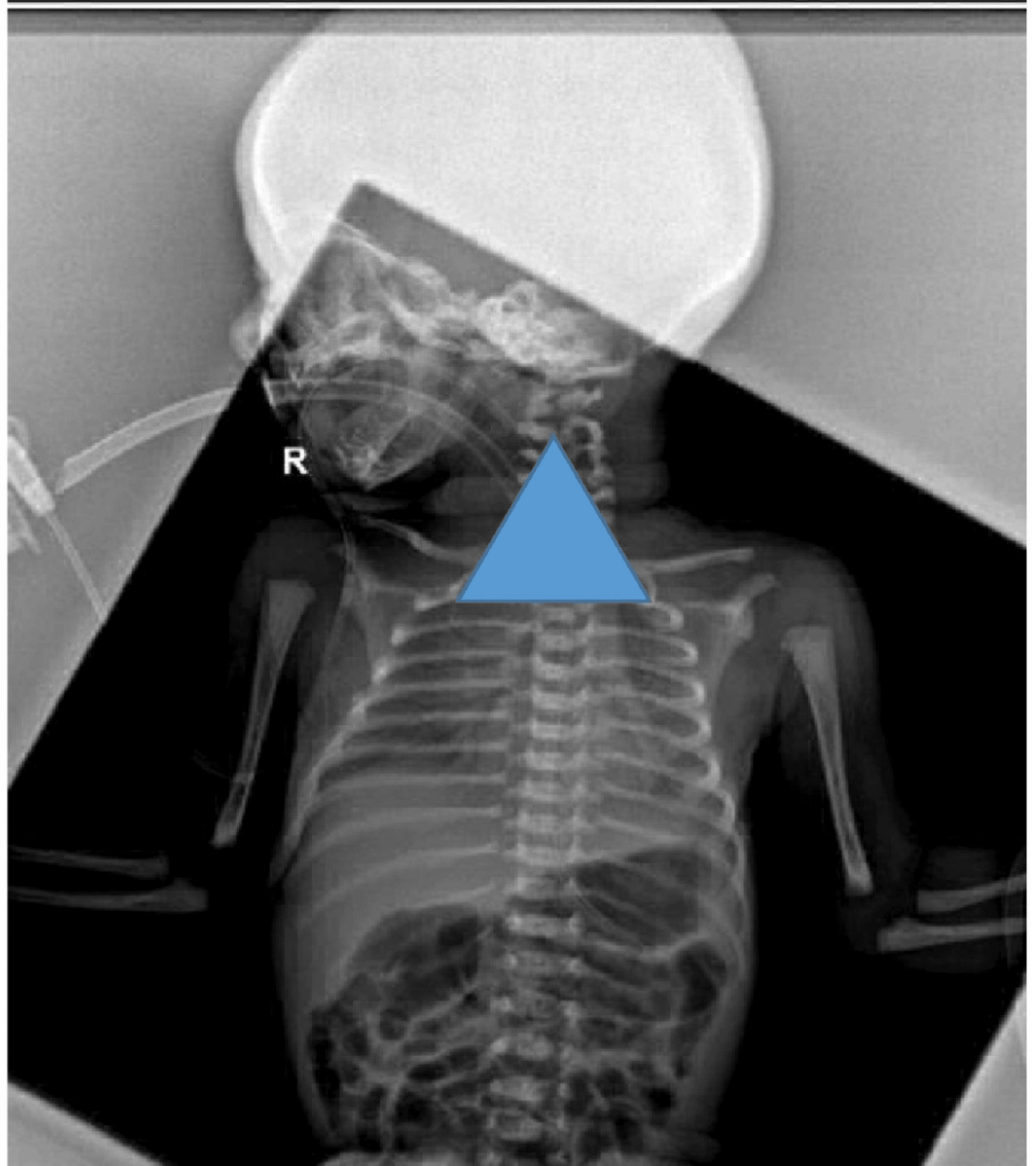
CXR image masking the ETT. CXR: chest x-ray, ETT: endotracheal tube

**Figure 2:**
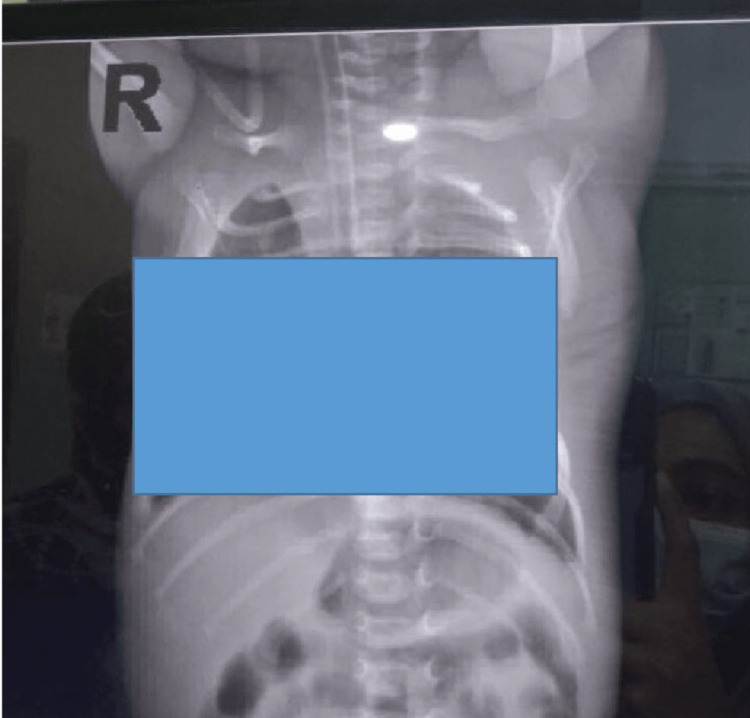
CXR image masking the lung fields. CXR: chest x-ray

**Figure 3:**
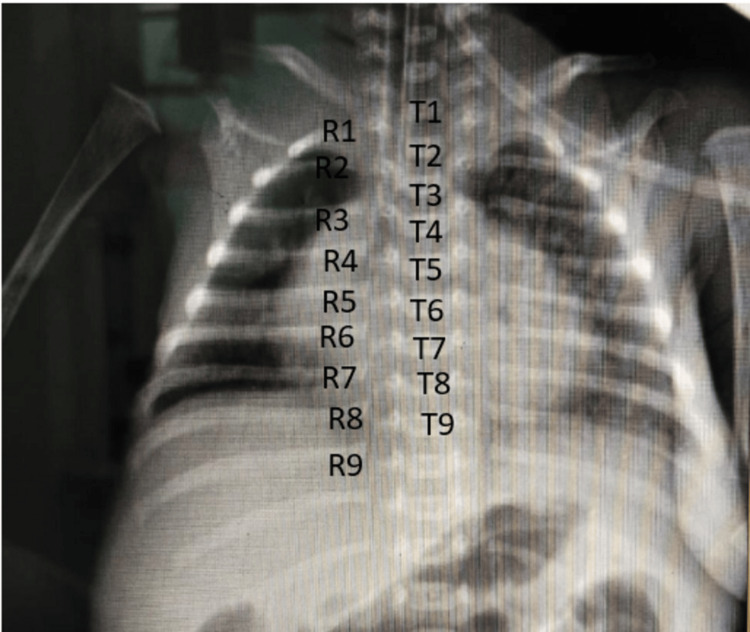
CXR image identification of vertebral levels for ETT tip position evaluation. CXR: chest x-ray, ETT: endotracheal tube

The CXR images (Figures 1, 2) illustrate the methodology employed. Figure 1 demonstrates a standard CXR where the ETT position is masked, allowing for lung inflation assessment. In contrast, Figure 2 shows a CXR with the lung fields masked, enabling targeted evaluation of the ETT position. Additionally, Figure 3 visually represents the process used for determining the ETT tip position by identifying vertebral levels.

Lung aeration was evaluated using operational definitions: optimal lung inflation/aeration was defined as visible lung expansion up to eight or eight and a half ribs on CXR, and suboptimal lung inflation/aeration as lung expansion less than eight ribs [9,10].

The ETT tip position was evaluated by the radiologist on CXRs by counting the vertebral levels at the distal end of the ETT, categorizing the position as either T1-T2 or T3-T4. As demonstrated in Figure 3, the first rib (R1) serves as a crucial anatomical landmark for vertebral-level identification. By counting downward from the first rib, corresponding thoracic vertebrae (T1-T9) can be systematically located, allowing precise determination of the ETT tip position.

To ensure precision and reduce bias, data collection was overseen by the principal investigator and supported by experienced neonatologists and radiologists. Data analysis was performed using SPSS version 26 (IBM Corp., Armonk, NY), with continuous variables reported as means and standard deviations, and categorical variables as frequencies and percentages. Chi-square tests were used to assess the association between ETT tip position and lung aeration, with a p-value of <0.05 considered significant. To account for potential confounders, data were stratified by gestational age, and post-stratification chi-square tests were conducted to assess any significant differences in the association between ETT position and lung aeration.

## Results

Our result shows that out of 149 neonates, 60 (40.3%) were female and 89 (59.7%) were male. The majority were preterm infants (105, 70.5%), while 44 (29.5%) were term. CXR findings indicated that 124 neonates (83.2%) exhibited optimal lung aeration, and 25 neonates (16.8%) had suboptimal lung aeration. Regarding ETT positioning, the tip was located at T1-T2 in 86 neonates (57.7%) and at T3-T4 in 63 neonates (42.3%). These results describe the distribution of lung aeration and ETT positioning but do not provide a direct comparison or statistical analysis of their association (Table 1).

**Table 1:** Comparison of neonatal characteristics and lung aeration by ETT tip position. ‡ Fisher's Exact, ∫Pearson Chi-square. All the data were expressed in n(%). ETT: endotracheal tube

	Overall	T1-T2 (n=86)	T3-T4 (n=63)	P-value
Gender				
Male	89(59.7)	47(54.7)	42(66.7)	0.146 ∫
Female	60(40.3)	39(45.3)	21(33.3)	
Gestational age				
Term	44(29.5)	28(32.6)	16(25.4)	0.345 ‡
Preterm	105(70.5)	58(67.4)	47(74.6)	
Gestational weight category				
Normal birth weight	24(16.2)	15(17.4)	9(14.5)	0.784 ‡
Low birth weight	62(41.9)	33(38.4)	29(46.8)	
Very low birth weight	42(28.4)	26(30.2)	16(25.8)	
Extremely low birth weight	20(13.5)	12(14)	8(12.9)	
CXR finding				
Optimal lung aeration	124(83.2)	74(86)	50(79.4)	0.283 ∫
Suboptimal lung aeration	25(16.8)	12(14)	13(20.6)	

Comparison of ETT tip positions

**Figure 4:**
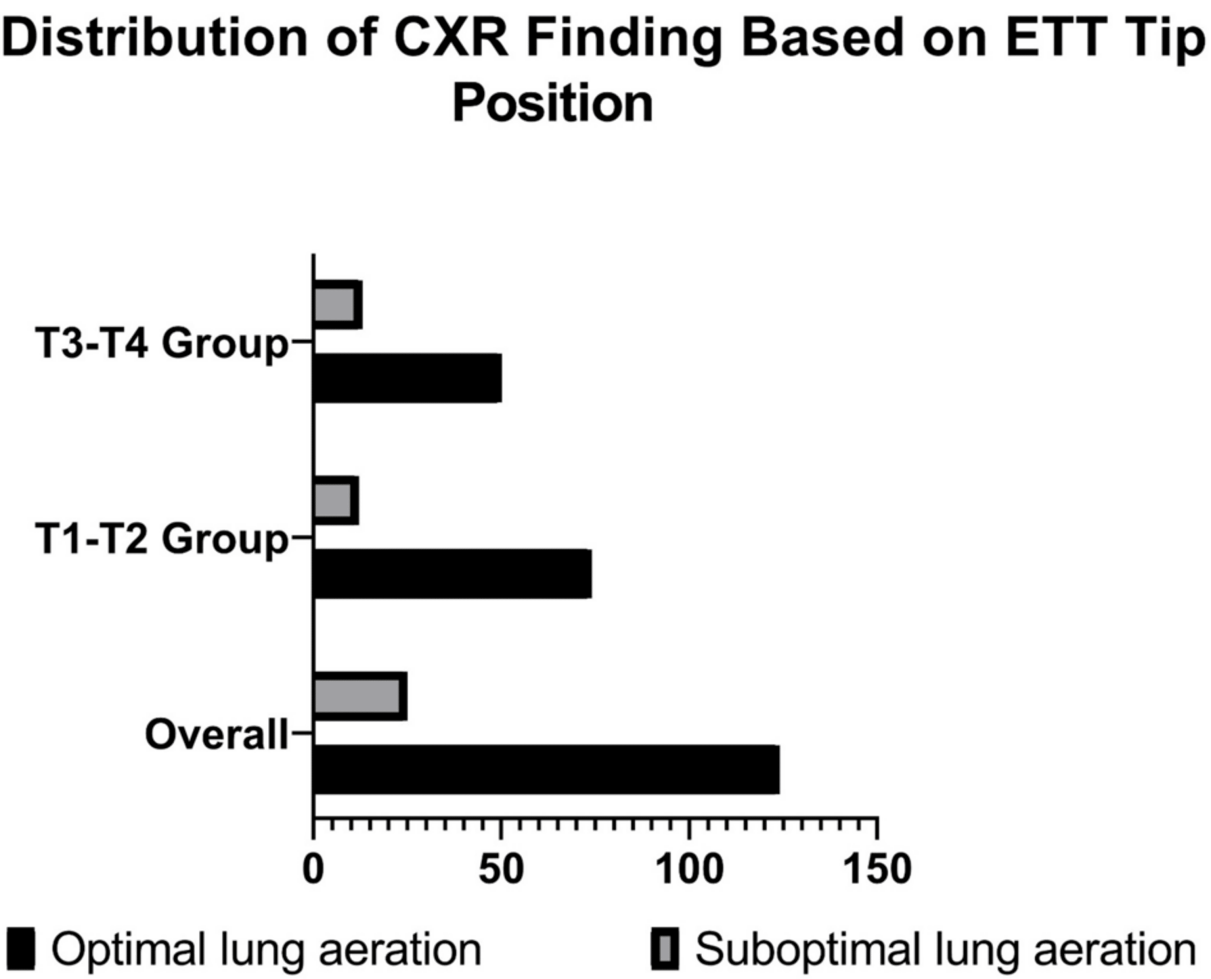
Distribution of CXR finding based on ETT tip position. CXR: chest x-ray, ETT: endotracheal tube

For lung aeration, 74 (86%) of neonates with the ETT tip at T1-T2 had optimal lung aeration, whereas 50 participants (79.4%) of those with the ETT tip at T3-T4 achieved optimal aeration (Figure 4). The p-value of 0.283 suggests that the variation in lung aeration between the two ETT positions is not statistically significant. Overall, the comparison shows no significant differences in gender distribution, gestational age, or lung aeration based on ETT tip position (Table 1).

Table 2 presents the comparison of neonatal characteristics and lung aeration between term (n=44) and preterm (n=105) neonates. Regarding the position of the ETT tip, 63.6% of term neonates had the ETT tip positioned between T1 and T2, while 55.2% of preterm neonates had the same positioning, again showing no significant difference (P = 0.347). CXR findings revealed optimal lung aeration in 81.8% of term neonates and 83.8% of preterm neonates, with suboptimal aeration in 18.2% and 16.2%, respectively. This difference was also not statistically significant (P = 0.768). Overall, Table 2 demonstrates that there were no significant differences in these neonatal characteristics between the term and preterm groups. 

**Table 2:** Comparison of neonatal characteristics and lung aeration by gestational age category. ‡ Fisher's Exact, ∫Pearson Chi-square. All of the data were expressed in n(%). ETT: endotracheal tube

	Term (n=44)	Preterm (n=105)	P-value
Gender			
Male	25(56.8)	64(61)	0.634 ∫
Female	19(43.2)	41(39)	
ETT tip position			
T1-T2	28(63.6)	58(55.2)	0.347 ∫
T3-T4	16(36.4)	47(44.8)	
CXR finding			
Optimal lung aeration	36(81.8)	88(83.8)	0.768‡
Suboptimal lung aeration	8(18.2)	17(16.2)	

Table 3 compares the characteristics of term and preterm neonates based on the position of the ETT tip and CXR findings. Among term neonates who had the ETT tip at the T1-T2 level, 72.2% exhibited optimal lung aeration, while 25% had suboptimal aeration. In contrast, among those with the ETT tip at the T3-T4 level, only 27.8% had optimal aeration, with 75% showing suboptimal aeration. This difference was statistically significant (P = 0.019), indicating that the position of the ETT tip influences lung aeration in term neonates.

**Table 3:** Comparison of term neonates' characteristics by ETT tip position and CXR finding. *P-value<0.05, ‡ Fisher's Exact, ∫Pearson Chi-square. All the data were expressed in n(%). ETT: endotracheal tube, CXR: chest x-ray

Gestational age category	ETT tip position	Chest x-ray finding		Total	P-value
		Optimal aeration	Sub-optimal Aeration		
Term	T1-T2	26(72.2)	2(25)	28(63.6)	0.019*‡
	T3-T4	10(27.8)	6(75)	16(36.4)	
	Total	36(100)	8(100)	44(100)	
Preterm	T1-T2	48(54.5)	10(58.8)	58(55.2)	0.745∫
	T3-T4	40(45.5)	7(41.2)	47(44.8)	
	Total	88(100)	17(100)	105(100)	

For preterm neonates who had the ETT tip at the T1-T2 level, 54.5% had optimal aeration, and 58.8% had suboptimal aeration. Among those with the ETT tip at the T3-T4 level, 45.5% showed optimal aeration, and 41.2% had suboptimal aeration. However, unlike in term neonates, this difference was not statistically significant (P = 0.745), suggesting that the ETT tip position does not significantly impact lung aeration in preterm neonates.

**Figure 5:**
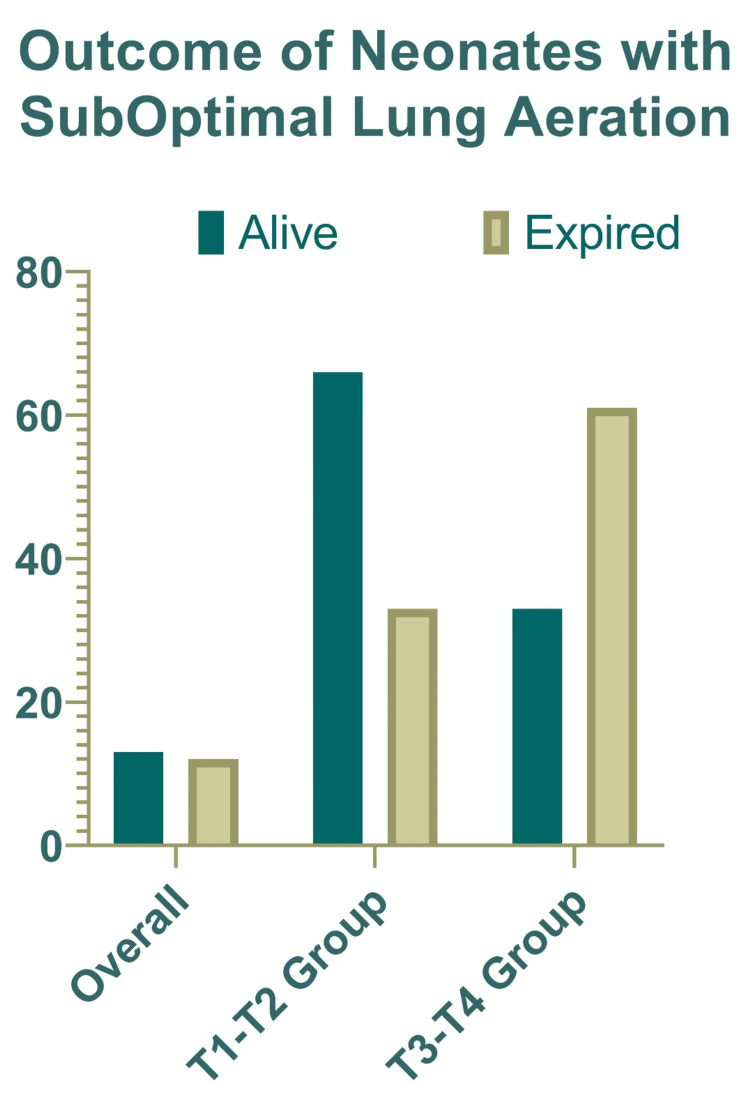
Outcome of neonates with suboptimal lung aeration.

Thirteen neonates survived in the poor lung aeration group; 66.7% of them had their ETT positioned between the T1 and T2 levels, and 38.5% had it positioned between the T3 and T4 levels. The ETTs of 33.3% and 61.5% of the 12 neonates who passed away were positioned at the T1-T2 and T3-T4 levels, respectively (Figure 5).

## Discussion

Identifying the best posture for EI is critical for safe airway management in small infants, such as neonates and preterm babies. Compared to adults, these patients have shorter tracheal lengths and reduced reserve capacity leading to hypoxia. The ideal strategy for determining the ETT insertion length for attaining optimal placement in newborns is still debated. The objective of this study was to determine the association between ETT position and optimal lung aeration in neonates admitted to the NICU at tertiary care centers in Karachi, Pakistan.

Our study involved 149 neonates, where 59.7% were male and 40.3% were female. The population was predominantly preterm infants (70.5). In terms of ETT tip positioning, 57.7% of neonates had the tip positioned between T1 and T2, while 42.3% had it at T3-T4. The percentage of neonates with the ETT positioned outside the T1-T2 range (53%) was consistent with findings from other studies, where this placement is considered suboptimal or incorrect [11-13].

This study revealed that the distribution of ETT tip positioning across different gestational weight categories did not show any statistically significant differences (p = 0.78). For instance, 17.4% of neonates with normal birth weight had the ETT tip at T1-T2, while 14.5% had it at T3-T4. Similarly, in the low-birth-weight group, 38.4% had the ETT at T1-T2 and 46.8% at T3-T4, with similar patterns observed in the very low and extremely low birth weight categories. These results are consistent with the findings of Flinn et al. [14], who concluded that there was no significant difference in the proportion of correctly placed ETTs using weight-based guides. However, contrastingly, some other studies suggest that body weight is highly correlated with tracheal length and ETT position in neonates [11,15]. Optimal lung aeration was achieved in 83.2% of the neonates, while the remaining 16.8% exhibited suboptimal lung aeration. This result contrasts with the findings of Thayyil et al. [3], who reported a higher rate of suboptimal lung aeration in 37.8% of neonates. The discrepancy between the two studies might reflect differences in nursing care quality, tube handling techniques, and overall clinical management.

Further analysis was conducted to compare the characteristics of term and preterm neonates concerning ETT tip position and lung aeration. Lung aeration differences between term (81.8% optimal) and preterm (83.8% optimal) neonates were also not significant (p = 0.76). These results suggest that both term and preterm neonates can achieve similar lung aeration outcomes irrespective of their developmental stage, provided the ETT is correctly placed. This finding contrasts with results from Thayyil et al. [3], who observed nonuniform lung aeration in 15% of extremely premature infants with optimal ETT positioning (group A) compared to 51% in those with suboptimal ETT positioning (group B), a significant difference (p = 0.01). Similarly, Pereira et al. reported that uneven inflation/aeration of the lungs was significantly higher in neonates with suboptimal ETT (above T1 and below T2) positioning, with 7% of uneven lung inflation in the suboptimal ETT placement group compared to 0% in the group with optimal positioning [16].

Our results reveal the critical impact of ETT position on lung aeration, particularly in term neonates. Specifically, term neonates with the ETT tip positioned at T1-T2 exhibited a significantly higher rate of optimal lung aeration (72.2%) compared to those with the tip at T3-T4 (27.8%), with the difference being statistically significant (p = 0.019). This finding underscores the importance of precise ETT placement at T1-T2 for ensuring optimal lung inflation in term neonates. In contrast, among preterm neonates, no significant difference in lung aeration was observed between the two ETT positions (p = 0.745), suggesting that factors beyond ETT positioning may influence lung aeration outcomes in this group.

These findings align with the study by Yamamoto and Schindler, which highlighted the strong correlation between body height and tracheal length in pediatric patients, including neonates [17]. This correlation between body height and tracheal length further emphasizes the need for individualized approaches to ETT placement, particularly in neonates, where small variations in tracheal length due to height differences can significantly impact the optimal positioning of the ETT and, consequently, lung aeration. Our results, particularly in term neonates, support the critical role of precise ETT positioning, which should be informed by accurate assessments of tracheal length, likely influenced by body height.

Several limitations should be noted in this study. First, the retrospective nature of the study limits the ability to establish causality, and unmeasured confounding variables, such as the skill level of the intubating personnel and variations in mechanical ventilation settings, were not controlled. Additionally, one limitation is the lack of documentation regarding neck positioning at the time of the CXRs. Another limitation is the absence of data on accidental extubations, which could not be assessed within the scope of this study. Whether the T1 position is associated with a higher risk of such events could only be determined through a prospective study. Future research should aim to validate these findings in larger cohorts and investigate the longitudinal impact of ETT positioning on respiratory outcomes in neonates.

## Conclusions

In conclusion, our study found a significant association between ETT tip positioning at T1-T2 and optimal lung aeration in term neonates. This suggests that precise ETT placement may play an important role in achieving better lung aeration in term infants, while slight positional deviations may be less impactful for lung aeration in preterm neonates. These findings may guide NICU protocols to consider gestational age when tailoring ventilation strategies, emphasizing the importance of anatomical and physiological differences in neonatal respiratory care.
